# Green citrate sol–gel synthesis of CuMn₂O₄/Dy₂O₃ nanocomposite for efficient separation, preconcentration, and determination of Cd(II) in food and environmental samples

**DOI:** 10.1007/s10661-026-15625-2

**Published:** 2026-07-01

**Authors:** Mustafa Soylak, Abdirashid Adam Isak, Furkan Uzcan, Orhan Turkoglu

**Affiliations:** 1 Faculty of Science, Chemistry Department, Erciyes University, 38039 Kayseri, Turkey; 2 Technology Research and Application Center (TAUM), Erciyes University, 38039 Kayseri, Turkey; 3 Turkish Academy of Sciences (TUBA), Ankara, Turkey; 4 Graduate School of Natural and Applied Sciences, Chemistry Graduate Program, Erciyes University, Kayseri, Turkey; 5 Department of Chemistry, Faculty of Sciences, Somali National University, Mogadishu, Somalia

**Keywords:** Green synthesis, CuMn₂O₄/Dy₂O₃ nanocomposite, Extraction, Cadmium, Food safety analysis

## Abstract

A novel CuMn₂O₄/Dy₂O₃ nanocomposite was synthesized and applied as an efficient adsorbent for the trace Cd(II) using micro solid-phase extraction (µSPE) coupled with high-resolution continuum source flame atomic absorption spectrometry (HR-CS-FAAS). The nanocomposite was prepared via a green, facile citrate sol–gel route followed by thermal annealing and was extensively characterized by FT-IR, XRD, SEM–EDX, BET, and TGA analyses. Key extraction parameters, including pH, adsorbent dosage, contact time, eluent type, and sample volume, were systematically optimized. Under the optimized conditions (pH 7.0, 7.5 mg adsorbent dosage, 1.0 mL of 1.0 M HNO₃ as eluent, and 40.0 mL of sample volume), the method achieved a preconcentration factor of 40. The procedure exhibited an instrumental LOD of 5.2 µg L⁻^1^, corresponding to a method LOD of 0.13 µg L⁻^1^ after accounting for the preconcentration factor of 40 below both the WHO and US EPA drinking water guideline values for cadmium together with high selectivity in the presence of competing ions. The analytical accuracy and robustness of the method were validated using certified reference materials. Its applicability was successfully demonstrated with commercial chocolate, juice, and wastewater samples.

##  Introduction

Heavy metals, including cadmium, pose serious health risks to people and other living things due to their hazardous properties (Behbahani et al., 2015; Dhaliwal et al., 2022; Koçoğlu et al., 2025; Mohammadi et al., 2026). In particular, cadmium and other naturally close heavy elements are considered among the most dangerous poisonous metals, and cadmium in particular causes acute toxicity that manifests as kidney damage, hypertension, and red blood cell loss (Mukherjee et al., 2023; Rustemli & Tuncay, 2026; Wu et al., 2022). Whenever exposed through inhalation or consumption of tainted food and water, the buildup of cadmium in humans’ biological systems over time intensifies its toxicity, leading to serious health problems, particularly in the kidneys and bones (Nordberg et al., 2018; Qing et al., 2021).

Cadmium is primarily found to be an impurity in ores containing other metals such as zinc, lead, and copper, and notably available as cadmium sulfide (CdS), and less than 1% of these ores are composed of cadmium (Haridy et al., 2025; Huang et al., 2025). However, the main industrial applications of cadmium are the production of nickel–cadmium (Ni–Cd) rechargeable batteries, which are mostly used in portable electric and electronics due to their high energy density and lifelong as well as electroplating, and using it as pigments in paints and ceramics, particularly the bright and weak colors from red through orange to yellow (Lee et al., 2021; Wang et al., 2023; Zhang & Reynolds, 2019). However, the usage of cadmium in many industrial applications has decreased significantly in recent years due to its toxicity and its resulting environmental hazards, notably among Ni–Cd batteries (Al Othman et al., 2013). Strict environmental regulations in many countries and the appearance of safer alternatives, such as lithium-ion batteries, have further contributed to this decline in the consumption of Ni–Cd batteries around the globe (Farias et al., 2024; Luo & Zhang, 2021). In the same context, cadmium-based compounds have also been used as stabilizers or additives in plastic production, but their application is declining due to similar health and environmental concerns (Roguz & Szaniawska, 2026; Turanlı & Gedik, 2021).

The US Environmental Protection Agency and the World Health Organization (WHO) have set specific levels for cadmium in drinking water at 0.005 mg/L and 0.003 mg/L, respectively, to limit the amount of cadmium allowed in drinking water (Briffa et al., 2020; Soylak et al., 2024). These standards are set to have the least possible influence on public health, especially for vulnerable groups and communities (Naksen et al., 2022). In addition to environmental and water quality measures, food safety standards also make clear that cadmium contamination, if it exceeds any specified limits, is unacceptable (Deng et al., 2025). For instance, in 2009, the panel on contaminants in the food chain of the European Food Safety Authority (EFSA) recommended lowering the PTWI to 2.5 µg/kg body weight, the tolerated weekly intake (TWI) limit (Mukherjee et al., 2023).

Ultrasound-assisted extraction (UAE), magnetic solid phase extraction (MSPE), and µSPE are essential as methods for determining, detecting, and analyzing heavy metals like cadmium (Shishov et al., 2023; Soylak et al., 2024). The methods used with these techniques are efficient, accessible, inexpensive, and they do not consume many numbers of materials (Soylak et al., 2024). Moreover, they are particularly advantageous in real sample analyses, where the presence of complex matrix components and the extremely low concentrations of target analytes pose significant challenges (Jalbani et al., 2016; Martins et al., 2025). These unique values are what make them relevant in complying with the environmental and food safety guidelines and in monitoring them, where precise actions are necessary when cadmium is at trace levels (Omidi et al., 2015).

µSPE is an easy analytical technique in terms of the separation, enrichment, and extraction of heavy metals, e.g., cadmium ions, from different samples like water and food products (Behbahani et al., 2018; Bojko, 2022). This technique has a particular privilege in terms of capturing cadmium selectively in a much greener method by not using toxic solvents and reducing the sample preparation time (Narin et al., 2002). In the µSPE method, a solid adsorbent is used to specifically capture cadmium ions from the sample matrix (Danala Danga et al., 2025). The sorbent is desorbed by shaking, centrifuging, and/or vortexing once the extraction is completed (Qezelje et al., 2026). The ability to achieve low detection limits for cadmium, minimal sample consumption, and reduced use of hazardous chemicals is among the µSPE’s many advantages (Badawy et al., 2022; Behbahani et al., 2014a, 2014b). However, among the limitations of this technique is that it could be affected by the type of sample matrix, and the sorbent material’s resistance may differ based on the cadmium ion concentration, as well as the sample type being analyzed (Keerthana et al., 2024). For those reasons, careful optimization of the µSPE conditions has immense importance to get reliable, precise, and accurate results (Behbahani et al., 2014a, 2014b; Williams et al., 2023).

At normal conditions, a noticeable number of chemicals belonging to the AB_2_X_4_ family, especially oxides (X = Oxygen), crystallize in the spinel structure. CuMn_2_O_4_ is a metal oxide of the spinel type and consists of several chemical properties that make it capable for the usage of heavy metal extraction. The efficacy of CuMn_2_O_4_, as a sorbent material, is referred to its unique chemical characteristics as well as the enhancing effects of Dy_2_O_3_. It is useful for environmental treatment and monitoring applications in industrial pollution-affected regions due to its high reactivity and thermal stability, which again allow for the effective adsorption and separation of hazardous heavy metal ions (Yousef et al., 2021; Xu et al., 2012; Abolhasani & Behbahani, 2015).

Given the severe health risks posed by cadmium exposure, the limitations of conventional extraction and preconcentration approaches, and the increasing demand for sensitive, selective, and environmentally benign analytical methods, the development of advanced sorbent materials remains an urgent research priority (You et al., 2025). Spinel-type oxides such as CuMn₂O₄ offer exceptional structural and chemical stability, while their performance can be further enhanced by strategic modification with rare-earth oxides such as Dy₂O₃, which improve both reactivity and thermal resistance (Roy et al., 2025). Building on these advantages, the present study introduces a novel CuMn₂O₄/Dy₂O₃ nanocomposite synthesized via a green citrate sol–gel approach, followed by comprehensive characterization and application in micro solid-phase extraction (µSPE) for Cd(II). By integrating material innovation with environmentally sustainable methodology, this work not only provides a reliable and cost-effective tool for trace-level cadmium monitoring in food and environmental samples but also contributes to the broader field of green analytical chemistry, aligning with current regulatory demands and global sustainability goals (Roy et al., 2025). The CuMn₂O₄/Dy₂O₃ nanocomposite was selected as a potential sorbent material due to its high surface reactivity, abundance of active adsorption sites, and favorable structural characteristics that may enhance Cd(II) adsorption efficiency. These properties make the nanocomposite a promising candidate for solid-phase extraction applications involving trace cadmium determination in complex food and environmental matrices.

## Experimental work

### Instrumentation

A comprehensive suite of laboratory instruments was employed to ensure precise extraction and reliable quantification of Cd(II) in chocolate and water matrices. Sample homogenization was achieved using a Cyclone Vortex Mixer (Model 12665, Ankara, Türkiye), followed by centrifugation with a Nuve NF400 benchtop centrifuge (Nuve, Ankara, Türkiye) to remove particulate matter. Quantitative analysis of Cd(II) was performed with a high-resolution continuum source atomic absorption spectrometer (HR-CS FAAS, ContrAA 800, Analytik Jena AG, Jena, Germany), which served as the primary instrument for trace metal determination. The synthesized adsorbent nanomaterial was structurally and elementally characterized using Fourier Transform Infrared (FT-IR) spectroscopy (Thermo Nicolet 5700, Thermo Fisher Scientific, Waltham, MA, USA), while phase identification was confirmed via X-ray diffraction (XRD) using a BRUKER AXS D8 ADVANCE diffractometer (Bruker, Karlsruhe, Germany). Surface morphology and microstructural features were examined by field emission scanning electron microscopy (FE-SEM, ZEISS Gemini 550, Carl Zeiss AG, Oberkochen, Germany) coupled with energy-dispersive X-ray spectroscopy (EDX) for elemental mapping. Textural properties, including surface area, pore volume, and pore size distribution, were evaluated using the Brunauer–Emmett–Teller (BET) nitrogen adsorption–desorption method on a Micromeritics Gemini VII analyzer (Micromeritics Instrument Corp., Norcross, GA, USA).

Microwave-assisted digestion of certified reference materials (CRMs) and chocolate samples was carried out in a closed-vessel system (Ethos Lean-1, Milestone Srl, Sorisole, Bergamo, Italy), enabling complete mineralization of organic matrices under controlled temperature and pressure conditions. Sample mass measurements were conducted using an analytical balance (RADWAG AS 22.C2.R, RADWAG, Radom, Poland) with a sensitivity of 0.1 mg, and pH values of working solutions were monitored with a calibrated digital pH meter (Hanna Instruments HI2001-02, Woonsocket, RI, USA).

### Reagents

All chemical reagents used throughout the study were of analytical reagent grade and procured from certified suppliers to ensure consistency and reproducibility of the experimental results. Deionized water with a resistivity of 18.2 MΩ·cm, obtained from a Milli-Q Direct 18 purification system (Millipore, Bedford, MA, USA), was used in all experiments for solution preparation and sample dilution. A certified cadmium standard solution, supplied by Labsert Chemical Reference Standard (Mersin, Türkiye), was employed for the preparation of Cd(II) stock solutions. Additional reagents, including copper(II) nitrate trihydrate (Cu(NO₃)₂·3H₂O), manganese(II) nitrate tetrahydrate (Mn(NO₃)₂·4H₂O), citric acid monohydrate (C₆H₈O₇·H₂O), dysprosium(III) oxide (Dy₂O₃), polyvinylpyrrolidone (PVP), ethylene glycol, concentrated nitric acid (HNO₃), hydrochloric acid (HCl), and hydrazine hydrate (N₂H₄·H₂O), were purchased from Sigma-Aldrich (St. Louis, MO, USA). Buffer solutions covering a pH range of 2.0–10.0 were prepared using high-purity reagents to maintain and control the pH conditions during the experiments. Phosphate buffer systems were used for pH values of 2.0, 3.0, 7.0, and 8.0; acetate buffers for pH 4.0, 5.0, and 6.0; and ammonium buffer for pH 9.0. All buffers were prepared following standard protocols to ensure stability and reproducibility. To verify the accuracy, traceability, and quality assurance of Cd(II) detection and quantification, certified reference materials (CRMs), namely ERM-CA713 (wastewater matrix) and ERM-BD512 (dark chocolate matrix), were analyzed. These CRMs were obtained from the European Commission’s Joint Research Centre (JRC, Geel, Belgium).

### Synthesis of CuMn₂O₄/Dy₂O₃

**Fig. 1:**
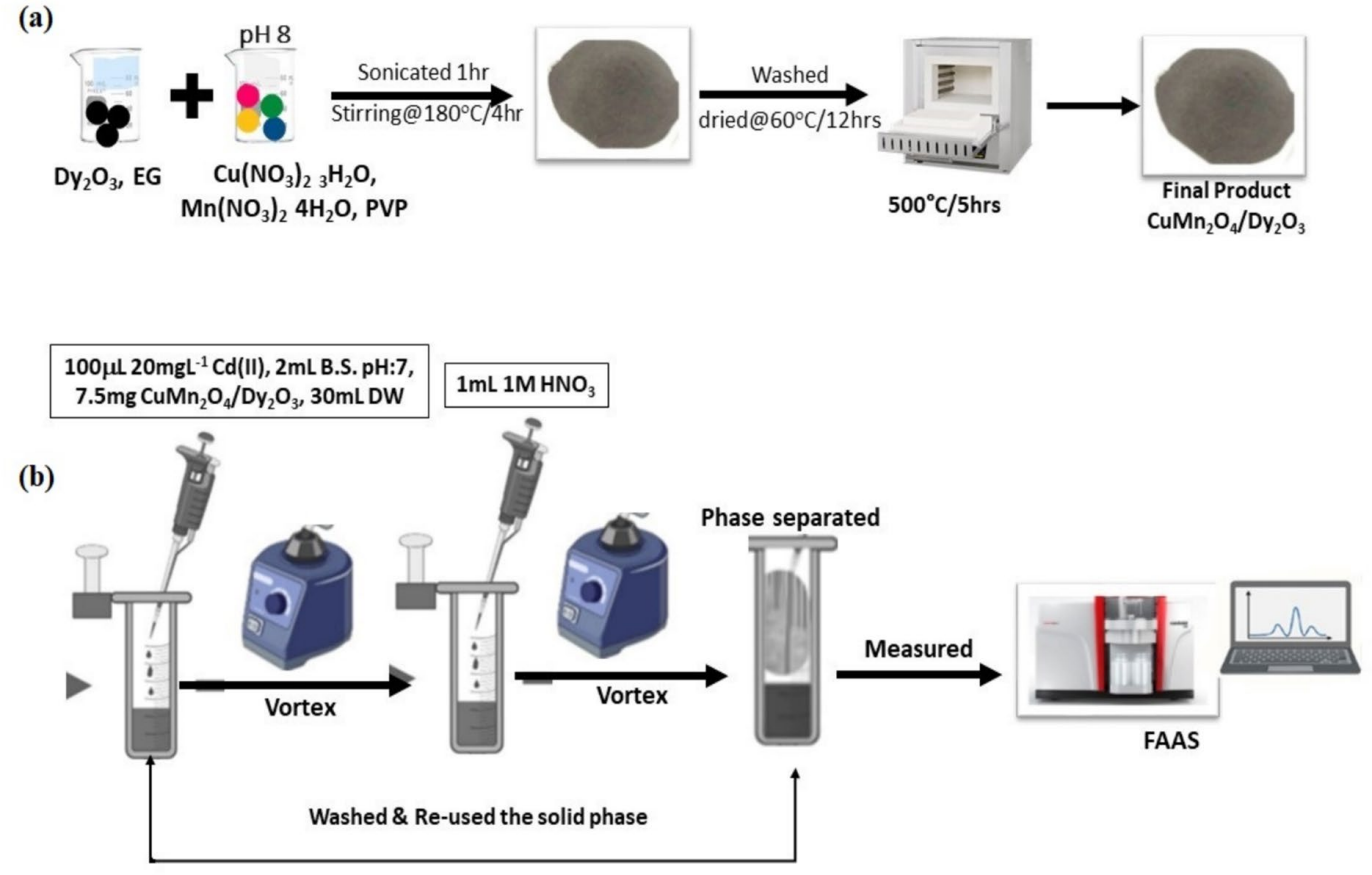
Diagrammatic overview of CuMn₂O₄/Dy₂O_3_ synthesis (**a**) and the experimental procedure for Cd (II) extraction (**b**)

The CuMn₂O₄/Dy₂O₃ nanocomposite was synthesized using a modified citrate sol–gel method followed by thermal annealing. Initially, 0.25 g of dysprosium (III) oxide (Dy₂O₃) was dispersed in 60 mL of ethylene glycol under continuous stirring to obtain a stable colloidal suspension. In a separate step, 1.55 g of copper(II) nitrate trihydrate (Cu(NO₃)₂·3H₂O), 2.50 g of manganese(II) nitrate tetrahydrate (Mn(NO₃)₂·4H₂O), 0.67 g of citric acid monohydrate (C₆H₈O₇·H₂O), and 0.40 g of polyvinylpyrrolidone (PVP) were dissolved in 40 mL of ultrapure water with vigorous stirring until complete dissolution. The pH of this precursor solution was adjusted to 8.0 by the slow, dropwise addition of 10 mL of hydrazine hydrate (N₂H₄·H₂O), which acted both as a reductant and as a pH modifier. Subsequently, the Dy₂O₃ dispersion was added to the metal–citrate–PVP solution, and the resulting mixture was sonicated for 1 h to enhance homogeneity and nanoscale dispersion. The mixture was then subjected to magnetic stirring at 180 °C for 4 h to promote gelation and initiate nanocomposite precursor formation. The obtained dark-colored precipitate was separated by centrifugation, thoroughly washed with distilled water and ethanol to eliminate residual ions and organic matter, and dried under vacuum at 60 °C for 12 h. Finally, the dried powder was annealed in a muffle furnace at 500 °C for 5 h in air to obtain the crystalline CuMn₂O₄/Dy₂O₃ nanocomposite (Fig. 1a) (Nolly et al., 2022).

### μSPE procedure

The micro solid-phase extraction (µSPE) of Cd(II) was carried out using CuMn₂O₄/Dy₂O₃ nanocomposite as the sorbent material (Fig. 1b). A Cd(II) solution (20 mg L⁻^1^) was prepared from a certified cadmium stock solution, and a 100 µL aliquot was transferred into a 50 mL centrifuge tube containing 7.5 mg of the CuMn₂O₄/Dy₂O₃ nanocomposite. To establish optimal extraction conditions, 2.0 mL of a pH 7.0 buffer solution was added to maintain a suitable environment for Cd(II) adsorption, and the final sample volume was adjusted to 40.0 mL with distilled water. The suspension was vortexed for 30 s to ensure homogeneous dispersion and facilitate the interaction between Cd(II) ions and the adsorbent surface. Following this, the mixture was centrifuged to separate the solid and liquid phases, and the supernatant was discarded. The retained adsorbent was treated with 1.0 mL of 1.0 M HNO₃ as an eluent to desorb Cd(II). The mixture was vortexed for 30 s and centrifuged for 60 s, and the resulting supernatant containing the eluted Cd(II) was collected in 15 mL tubes for quantification by flame atomic absorption spectrometry (FAAS). For reusability studies, the exhausted adsorbent was regenerated by sequential washing: three times with 3.0 M HNO₃ to remove residual metal ions, followed by single rinses with distilled water and ethanol. The material was then dried in a vacuum oven at 60 °C for 12 h prior to reusing. Although the regeneration procedure indicated the potential reusability of the CuMn₂O₄/Dy₂O₃ nanocomposite, detailed long-term adsorption/desorption cycle studies and post-regeneration structural characterization were not investigated in the present work and should be addressed in future studies.

### Applications

To evaluate the applicability and robustness of the developed method, analyses were performed on real samples, including wastewater, juice, and commercially available chocolate as representative food matrices. Prior to analysis, chocolate samples and chocolate CRM were microwave-assisted acid-digested to ensure complete mineralization of organic components. Approximately 0.25 g of homogenized chocolate was weighed into Teflon digestion vessels, followed by the addition of 5 mL concentrated HNO₃ and 2 mL H₂O₂. Digestion was carried out in a closed-vessel microwave system at 180 °C for 30 min under controlled pressure conditions. After cooling, the digests were transferred and diluted to a final volume of 40.0 mL with deionized water. The pH of both digested chocolate and wastewater samples was adjusted to 7.0 prior to extraction. Method accuracy and potential matrix effects were evaluated through spike recovery experiments. Known concentrations of Cd(II) were spiked into both wastewater and digested chocolate samples at levels of 10 and 25 µg·L⁻^1^, along with unspiked controls. Each concentration was analyzed in triplicate. Recovery values were calculated by comparing measured concentrations with theoretical values, yielding satisfactory recoveries within acceptable analytical limits. These results confirmed the reliability and suitability of the developed µSPE–FAAS method for trace-level cadmium determination in complex matrices.

## Results and discussions

### Characterization of the adsorbent

**Fig. 2:**
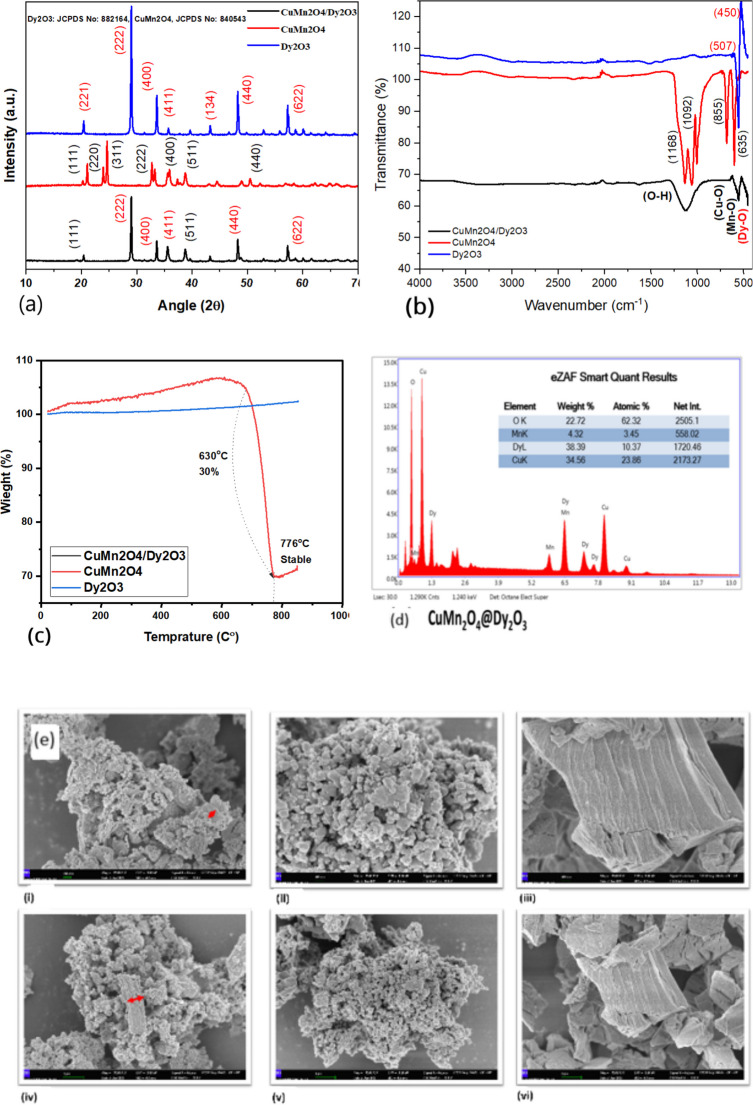

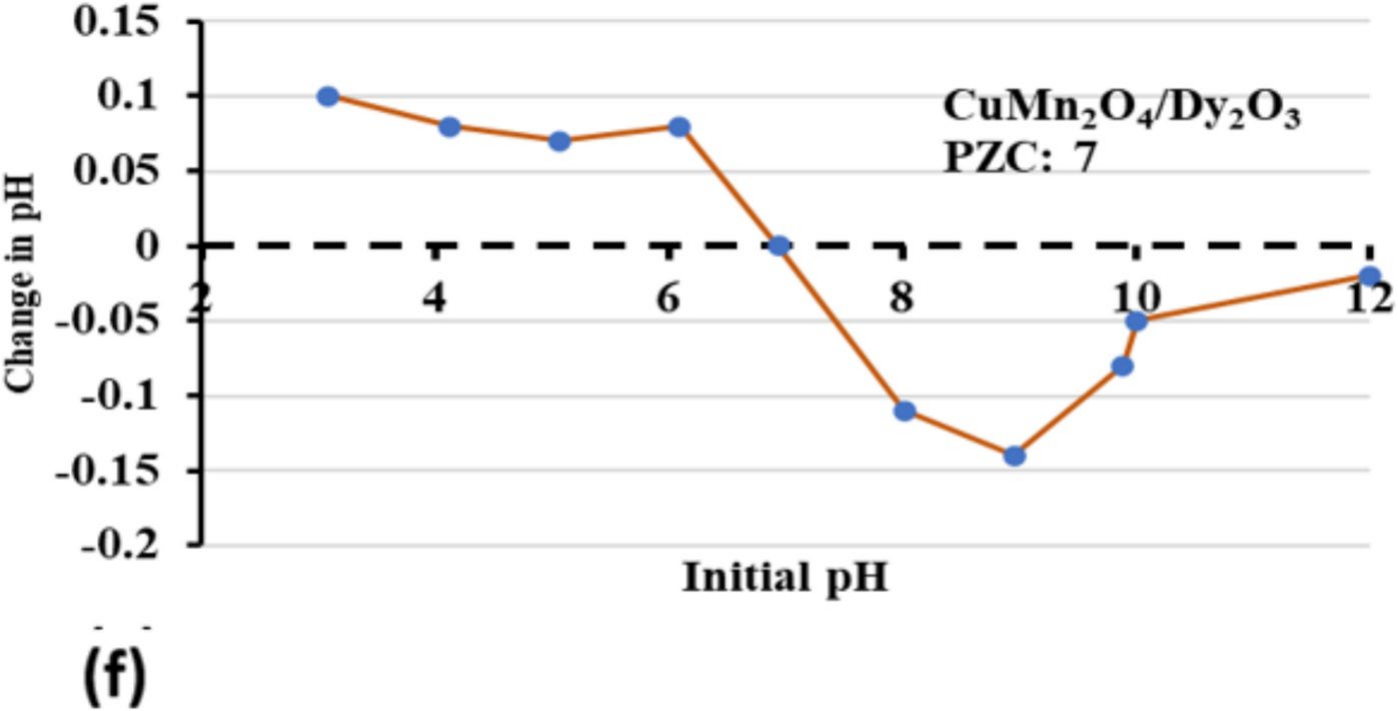
**a** XRD. **b** FT-IR. **c** TGA. **d** Elemental percentages of CuMn_2_O_4_/Dy_2_O_3_ nanocomposite. **e** SEM micrographs of the synthesized materials at different magnifications: 200 nm, 20 k × CuMn₂O₄/Dy₂O₃ nanocomposite (i); 200 nm, 20 k × CuMn₂O₄ (ii); 200 nm, 20 k × Dy₂O₃ (iii); 1 µm, 10 k × CuMn₂O₄/Dy₂O₃ nanocomposite (iv); 1 µm, 10 k × CuMn₂O₄ (v); 1 µm, 10 k × Dy₂O₃ (vi). The images reveal the surface morphology, porosity, and particle aggregation of the composites, highlighting the effect of Dy₂O₃ doping on particle coarsening and structural uniformity. **f** PZCs test: (pH 2.0–12, 20 mL of 0.1 M NaNO_3_, 7.5 mg of CuMn_2_O_4_/Dy_2_O_3_)

The XRD pattern of the CuMn₂O₄/Dy₂O₃ nanocomposite confirmed the successful formation of a crystalline spinel phase. The sharp and well-defined diffraction peaks observed at 2θ values corresponding to the (220), (311), (400), (511), and (440) planes matched the characteristic reflections of the spinel CuMn₂O₄ structure (JCPDS No. 34–1400) (Dhaliwal et al., 2022; Kazemi & Sobhani, 2023). Additional weak reflections associated with Dy₂O₃ were also detected, indicating the presence of the dopant phase without disrupting the primary spinel lattice (Fig. 2a).

The FTIR spectrum of the composite (Fig. 2b) further validated the structural composition and surface chemistry. Distinct absorption bands at 558 cm⁻^1^ and 635 cm⁻^1^ were assigned to metal–oxygen (M–O) stretching vibrations of Cu–O and Mn–O bonds within the spinel framework, confirming the formation of CuMn₂O₄. A broad band at 3440 cm⁻^1^ corresponded to O–H stretching vibrations, indicating the presence of adsorbed hydroxyl groups or surface water, while the band at 1630 cm⁻^1^ was attributed to H–O–H bending vibrations of physisorbed water molecules (Kazemi & Sobhani, 2023).

Thermogravimetric analysis (TGA) revealed a three-stage weight-loss profile (Fig. 2c). The initial weight loss of 3.8% below 150 °C was attributed to desorption of physisorbed water and surface hydroxyl groups. A second stage between 150 and 400 °C corresponded to the decomposition of residual organic moieties and precursor salts. Above 400 °C, the material exhibited remarkable thermal stability with negligible mass loss up to 700 °C, reflecting the robust crystallinity of the spinel structure. The overall weight loss of 30.4% indicated good thermal resilience, which is essential for adsorbent regeneration and reuse under elevated thermal conditions.

The BET nitrogen adsorption–desorption isotherm exhibited typical mesoporous characteristics. The BET surface area was measured as 1.522 m^2^·g⁻^1^, with a pore volume of 0.002 cm^3^·g⁻^1^ and an average pore diameter of 5.3 nm. These parameters confirmed the mesoporous nature of the material, which is favorable for rapid mass transfer and accessibility of adsorption sites. The incorporation of Dy₂O₃ contributed to surface texturing and reduced particle sintering, thereby preserving the available surface area (Altunay et al., 2024; Azooz et al., 2025).

EDX analysis confirmed the elemental composition of the CuMn₂O₄/Dy₂O₃ composite, with prominent peaks corresponding to Cu, Mn, O, and Dy, while elemental mapping demonstrated a uniform distribution of Dy throughout the matrix without any evidence of segregation (Fig. 2d). SEM micrographs revealed a moderately porous, agglomerated nanostructure composed of quasi-spherical particle clusters (Fig. 2e). The morphology exhibited interconnected pores and interparticle voids, facilitating rapid adsorbate diffusion. Notably, Dy₂O₃ doping effectively reduced particle coarsening, likely by influencing nucleation and growth kinetics during synthesis, which in turn enhanced both the surface area and structural stability of the composite.

### Effect of pH and point of zero charge (PZC)

**Fig. 3:**
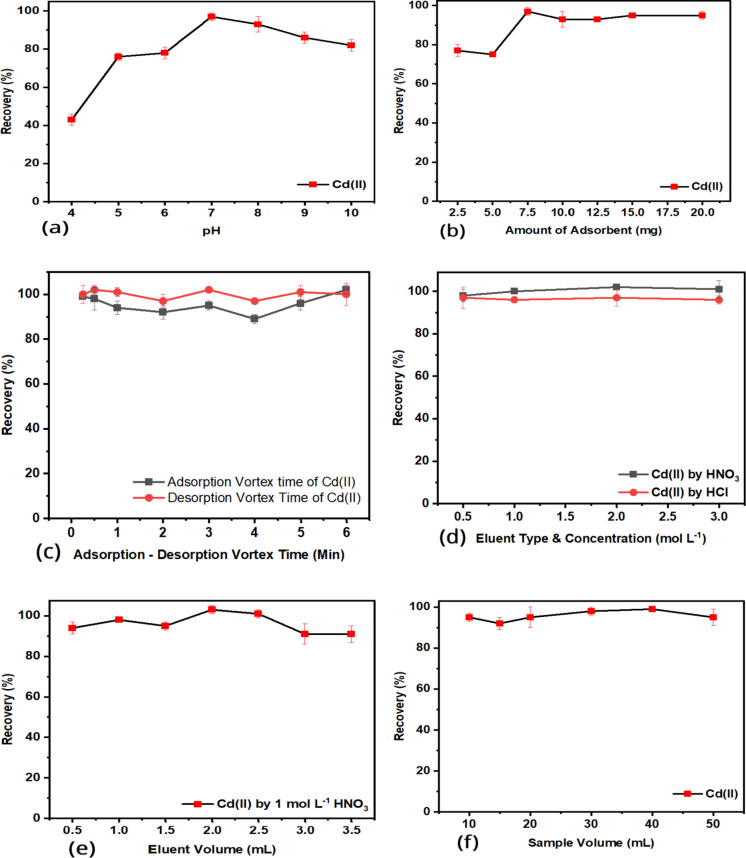
Optimization of experimental parameters for the micro-solid phase extraction of Cd(II) using CuMn₂O₄/Dy₂O₃ nanocomposite: effect of pH (**a**), adsorbent dosage (**b**), adsorption–desorption vortex time (**c**), eluent type and concentration (**d**), eluent volume (**e**), and sample volume (**f**). Experiments were conducted in triplicate (*N* = 3) under the following optimized conditions: pH 7.0, 2 mL buffer solution, 7.5 mg adsorbent, 40 mL sample volume, and elution with 1 mL 1 M HNO₃

The pH of the solution is a critical factor influencing the adsorption efficiency of heavy metal ions, as it governs both the speciation of metal ions in solution and the surface charge of the adsorbent (Salman et al., 2023; Yılmaz et al., 2025). The adsorption of Cd(II) onto the CuMn₂O₄/Dy₂O₃ nanocomposite was investigated within a pH range of 4.0–10. The recovery increased steadily from pH 4.0 to 7.0, reaching a maximum of 97% at pH 7.0. Beyond this point, the adsorption efficiency plateaued at pH 8.0 and subsequently declined at higher pH values (9.0–10). At strongly acidic conditions (pH 4), recoveries were below 50%, indicating weak interactions between Cd(II) and the adsorbent surface. The point of zero charge (PZC) of the composite was determined to be pH 7.0 (Fig. 2f), implying that the surface is positively charged at pH < 7.0 and negatively charged at pH > 7.0. The enhanced adsorption observed at pH 7.0 can be attributed to favorable electrostatic interactions and complexation processes occurring near the PZC (Chen et al., 2023). At lower pH values, extensive protonation of surface functional groups (–OH, –O⁻, Mn–O, Cu–O) limits the availability of binding sites and induces electrostatic repulsion between the positively charged surface and Cd^2^⁺ ions. As the pH approaches neutrality, surface deprotonation increases, creating a neutral or slightly negative surface environment that facilitates electrostatic attraction and complexation with divalent Cd^2^⁺ ions (Serencam et al., 2008). In addition, Cd(II) species exist predominantly as free Cd^2^⁺ around pH 7.0, ensuring high mobility and availability for adsorption (Antep et al., 2026; Ghaedi et al., 2013). The formation of inner-sphere complexes through coordination with surface oxygen and hydroxyl groups (Cu–O⁻, Mn–O⁻, Dy–OH), the latter introduced by the basic rare-earth oxide component further enhances affinity at this pH (Majid et al., 2025). At alkaline conditions (pH > 8.0), adsorption decreased due to the hydrolysis of Cd(II) and precipitation of Cd(OH)₂, which reduced the concentration of free Cd^2^⁺ in solution. Furthermore, the increasingly negative surface charge at high pH values repelled hydroxylated Cd species, further diminishing uptake. As shown in Fig. 3a, the optimal adsorption at pH 7 reflects a balance between surface charge compatibility, favorable ion speciation, and active site availability. Therefore, pH 7.0 was selected as the optimal condition for subsequent adsorption experiments.

### Effect of the adsorbent dosage

In this study, the effect of adsorbent dosage on Cd(II) recovery was systematically evaluated, as the amount of sorbent directly determines the number of available active binding sites for metal ion uptake (Chen et al., 2023). Experiments were performed with CuMn₂O₄/Dy₂O₃ dosages ranging from 2.5 to 20 mg under identical experimental conditions. The recovery efficiency increased with adsorbent mass, reflecting enhanced accessibility of active sites. At 2.5 mg, the recovery rate was 77%, which increased to 97% at 7.5 mg. Beyond this point, further increases in dosage (10–20 mg) resulted in only marginal improvements, with recoveries stabilizing around 95%. This plateau effect can be attributed to the saturation of adsorption sites and the establishment of equilibrium between Cd(II) ions and the available surface functional groups. Consequently, 7.5 mg was identified as the optimal dosage, offering maximum recovery with minimal adsorbent consumption. This optimized value was therefore employed in all subsequent experiments (Fig. 3b).

### Optimization of adsorption and desorption time

In addition to adsorbent dosage, the influence of contact time on both adsorption and desorption processes was systematically examined to identify the minimum time required for efficient interaction between Cd(II) ions and the CuMn₂O₄/Dy₂O₃ surface. Contact time is a critical kinetic parameter in adsorption studies, as it dictates the rate of analyte capture and release, thereby governing the overall efficiency and rapidity of the separation process. A series of experiments was conducted by varying both adsorption and desorption contact time from 10 s to 6 min, while maintaining constant pH, adsorbent dosage, and Cd(II) concentration. The results demonstrated that equilibrium was rapidly established, with maximum performance achieved at 30 s for both. At this point, Cd(II) adsorption reached 98% recovery, while desorption with nitric acid yielded 102% recovery (Fig. 3c). Extending the contact time beyond 30 s did not produce any significant improvement, confirming the rapid kinetics and high efficiency of the developed method.

### Optimization of eluent type, volume, and concentration

Another critical parameter in the method optimization was the establishment of suitable elution conditions to ensure efficient desorption of Cd(II) ions from the CuMn₂O₄/Dy₂O₃ nanocomposite. The optimization focused on three factors: the eluent type, its concentration, and the volume used during the desorption step (Ullah et al., 2024). Appropriate eluent selection is essential for achieving quantitative recovery of the target analyte while preserving the adsorbent’s structural integrity and reusability. Two mineral acids, nitric acid (HNO₃) and hydrochloric acid (HCl), were evaluated as eluents at concentrations ranging from 0.5 to 3.0 M, with volumes ranging from 0.5 to 3.5 mL. The results demonstrated that 1.0 mL of 1.0 M HNO₃ provided the most efficient performance, yielding complete desorption across eluent type and concentration, and 98% recovery with respect to eluent volume (Fig. 3d, e). The superior performance of HNO₃ compared to HCl can be attributed to its stronger oxidizing properties and enhanced ability to disrupt metal oxygen surface interactions, which minimizes residual binding and ensures quantitative Cd(II) recovery (Fufa et al., 2025). Based on these findings, 1.0 mL of 1.0 M HNO₃ was selected as the optimal elution condition and applied in all subsequent experiments.

### Sample volume

Sample volume is a critical parameter in extraction and preconcentration methodologies, as it directly governs the enrichment factor, detection sensitivity, and overall analytical performance (Altunay et al., 2026; Bahadir et al., 2018; Saracoglu et al., 2001). To evaluate its effect, sample volumes ranging from 10 to 50 mL were systematically tested under the previously optimized experimental conditions. The results demonstrated that a sample volume of 40 mL yielded the highest recovery efficiency, with 99% recovery of Cd(II), while simultaneously maintaining excellent reproducibility (Fig. 3f). Although volumes between 30 and 50 mL produced comparable recovery values, they were associated with slightly higher standard deviations, indicating reduced precision at both lower and higher volumes. The superior performance observed at 40 mL can be attributed to the optimal balance between sufficient analyte adsorbent interaction and minimized dilution effects, which collectively enhance both sensitivity and reliability. Based on these results, 40 mL was selected as the optimal sample volume for subsequent studies. Considering the optimized elution volume of 1.0 mL, the corresponding preconcentration factor was calculated to be 40, highlighting the strong potential of the developed CuMn₂O₄/Dy₂O₃-based µSPE method for trace-level Cd(II) determination.

### Matrix effects

An investigation of common coexisting ions was conducted to evaluate potential matrix effects on the selectivity and robustness of the developed micro-solid-phase extraction method. In real samples, a variety of foreign ions often compete with the target analyte, particularly during the adsorption–desorption step. Therefore, assessing the impact of these ions is crucial for validating the method’s reliability under realistic conditions. A range of potentially interfering cations and anions was examined, including Ca^2^⁺, Mg^2^⁺, Na⁺, K⁺, Fe^3^⁺, Ni^2^⁺, as well as Cl⁻, NO₃⁻, SO₄^2^⁻, and CO₃^2^⁻. As presented in Table 1 the recovery rates of Cd(II) in the presence of these ions ranged from 95 to 102%, confirming that none of the tested matrix components caused significant interference in either adsorption or desorption. These findings clearly demonstrate that the CuMn₂O₄/Dy₂O₃ adsorbent possesses high selectivity toward Cd(II), even in complex sample matrices.

**Table 1:** Effect of common coexisting ions on the adsorption of Cd(II) using CuMn₂O₄/Dy₂O₃ nanocomposite. Experiments were performed in triplicate (*N* = 3)

Ion	Salt	Concentration (mg L^−1^)	Recovery, %^a^
Na^+^	NaNO_3_	5000	97 ± 3
K^+^	KCl	5000	99 ± 2
Ca^2+^	CaCl_2_	1000	96 ± 4
Mg^2+^	MgCl_2_	1000	100 ± 2
Cl^−^	KCl	5000	99 ± 2
NO_3_^−^	NaNO_3_	5000	97 ± 3
SO_4_^2−^	Na_2_SO_4_	500	95 ± 2
CO_3_^2−^	Na_2_CO_3_	500	98 ± 2
Fe^3+^	FeCl_3_	5	99 ± 4
Ni^2+^	Ni(NO_3_)_2_	5	98 ± 5

^a^Mean ± standard deviation

**Fig. 4:**
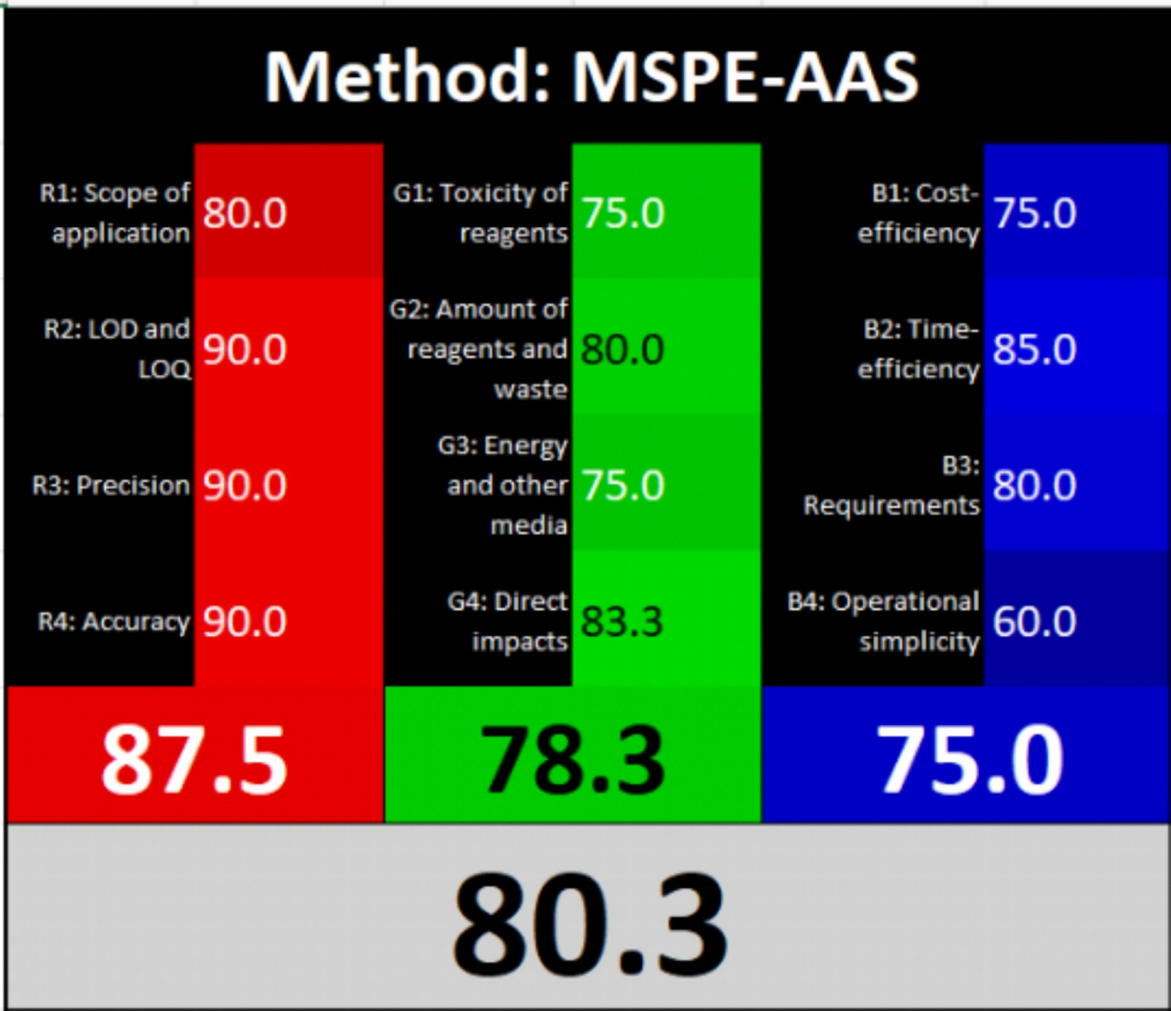
Whiteness assessment of the µSPE–FAAS method using CuMn₂O₄/Dy₂O₃ for Cd(II) determination. Scores for analytical performance (red), environmental impact (green), and practical efficiency (blue) are shown, with an overall whiteness score of 80.3%, demonstrating the method’s reliability and suitability for sustainable trace metal analysis

According to the whiteness assessment method reported in, the µSPE–FAAS procedure for Cd(II) determination using CuMn₂O₄/Dy₂O₃ as the adsorbent was evaluated using the Whiteness Analytical Criteria (WAC) (Nowak et al., 2021). The method demonstrated high analytical performance, achieving excellent precision, accuracy, and sensitivity, resulting in a strong red score of 87.5%. The environmental impact was moderate, with a green score of 78.3%, reflecting the reasonable use of reagents and low associated hazards, although the toxicity of some reagents and energy consumption remain areas for improvement. In terms of practical efficiency, the method achieved a blue score of 75.0%, particularly regarding time efficiency and cost-effectiveness, while operational simplicity (60%) could be further enhanced. Overall, as shown in Fig. 4, the method attained a comprehensive whiteness score of 80.3%, demonstrating its well-balanced, reliable, and sustainable applicability for trace-level Cd(II) determination in both food and environmental matrices.

### Analytical performance

The analytical performance of the CuMn₂O₄/Dy₂O₃/µSPE procedure was systematically evaluated for the separation and extraction of Cd(II) in commercial chocolate, juice, and water samples. The instrumental limit of detection (LOD) and limit of quantification (LOQ) were calculated according to the ICH guideline as LOD = 3.3 σ/s and LOQ = 10 σ/s, where σ is the standard deviation of the blank signal (*n* = 10) and s is the slope of the calibration curve. The method demonstrated high sensitivity, with instrumental LOD and LOQ values of 5.2 and 17.2 µg L⁻^1^, respectively. Taking the preconcentration factor of 40 into account, the corresponding method LOD and LOQ for the original sample matrices are 0.13 and 0.43 µg L⁻^1^, which lie well below both the WHO (3 µg L⁻^1^) and US EPA (5 µg L⁻^1^) drinking water guideline values for cadmium, confirming the applicability of the procedure to trace-level monitoring in environmental waters. The linear dynamic range extended from 17.2 to 1900 µg L⁻^1^ with excellent linearity (*R*^2^ = 0.994). Repeatability and reproducibility studies yielded relative standard deviations of 2.1% and 1.9%, with corresponding recoveries of 99.3% and 98.6%, respectively. The preconcentration and enrichment factors were 40 and 39.4, respectively. Together, these figures of merit confirm that the CuMn₂O₄/Dy₂O₃/µSPE procedure provides a reliable, accurate and highly sensitive platform for trace-level determination of Cd(II) in complex matrices.

### Applications

Two certified reference materials (CRMs) were analyzed to assess the accuracy and validity of the developed micro-solid phase extraction (µSPE) method using CuMn₂O₄/Dy₂O₃ as the adsorbent. These CRMs represented distinct and complex sample matrices: a solid matrix (ERM-BD512, dark chocolate, mg kg⁻^1^) and a liquid matrix (ERM-CA713, wastewater, µg L⁻^1^) to ensure the method’s broad applicability. The results presented in Table 2 demonstrate excellent agreement between the certified values and those obtained via the developed procedure, with recovery rates ranging from 99.8 to 100.9%. Statistical analysis using the *t*-test showed no significant difference between measured and certified values (*p* > 0.05), indicating the method’s reliability and accuracy for trace-level Cd(II) determination in complex matrices.

**Table 2:** Validation of the developed CuMn₂O₄/Dy₂O₃-based micro-solid phase extraction method using certified reference materials (CRMs). The table presents the certified and experimentally determined Cd(II) concentrations, recovery percentages, and T-test results at a 95% confidence level, demonstrating the accuracy and reliability of the method in both solid (dark chocolate) and liquid (wastewater) matrices

Certified reference materials (CRMs)	Certified value	Found value	Recovery, %	*T*-test
ERM-BD512 (dark chocolate, mg kg^−1^)	0.302 ± 0.013	0.305 ± 0.02	100.9 ± 1	Confidence level: 95% *p*-value = 0.0866 *T* table value = 2.228
ERM-CA713 (wastewater, µg L^−1^)	5.09 ± 0.20	5.08 ± 0.20	99.8 ± 0	

The applicability of the method was further evaluated using real-world samples, including wastewater, juice, and commercially available chocolate matrices. As summarized in Table 3, spike recovery experiments were conducted at 10 µg L⁻^1^ and 25 µg L⁻^1^ (or µg kg⁻^1^ for solid samples) Cd(II) levels. The recoveries were consistently high across all sample types, ranging from 90.4 to 108.0%, with low relative standard deviations (RSD ≤ 3%), confirming the method’s precision and reproducibility under realistic conditions. The slightly lower recovery obtained for Wastewater-1 (90.4%) and the relatively higher recovery observed for Chocolate-2 (108%) were attributed to matrix-induced suppression and enhancement effects, respectively, arising from the high inorganic content of wastewater and residual organic constituents present in the chocolate matrix during trace Cd(II) determination. Notably, unspiked samples indicated Cd(II) levels below or near the detection limit, highlighting the method’s capability for trace analysis in food and environmental samples. These findings demonstrate that the CuMn₂O₄/Dy₂O₃-based µSPE procedure provides a robust, accurate, and reproducible platform for Cd(II) determination in both liquid and solid matrices, suitable for routine monitoring and regulatory compliance.

**Table 3:** Application of the CuMn₂O₄/Dy₂O₃ adsorbent in real sample matrices (wastewater, fruit juice, and commercial chocolates) for the determination of Cd(II) using micro-solid phase extraction (*N* = 3)

Sample	Added (µg L^−1^)	Found (µg L^−1^) ^a^	Recovery %^b^
Wastewater-1	0	8.4 ± 0.012	––
	10	17.9 ± 0.013	95.0 ± 2
	25	31 ± 0.013	90.4 ± 2
Wastewater-2	0	9 ± 0.011	––
	10	19.1 ± 0.009	101.0 ± 3
	25	33 ± 0.009	96.0 ± 1
Wastewater-3	0	9 ± 0.012	––
	10	18.5 ± 0.015	95.1 ± 1
	25	33.4 ± 0.015	97.6 ± 3
Mixed fruit juice	0	10 ± 0.012	––
	10	19.4 ± 0.011	94.0 ± 3
	25	34.3 ± 0.019	97.2 ± 1
Sample	Added (µg kg^−1^)	Found (µg kg^−1^)	Recovery%
Chocolate-1	0	8.1 ± 0.012	––
	10	18.2 ± 0.010	101.0 ± 1
	25	32.3 ± 0.009	96.8 ± 0
Chocolate-2	0	7.4 ± 0.017	––
	10	18.2 ± 0.009	108.0 ± 3
	25	30.9 ± 0.012	94.4 ± 1
Chocolate-3	0	9.5 ± 0.012	––
	10	19.6 ± 0.009	101.0 ± 2
	25	34.1 ± 0.013	98.4 ± 4
Chocolate-4	0	9 ± 0.011	––
	10	18.3 ± 0.010	93.1 ± 1
	25	33.1 ± 0.014	96.4 ± 2
Chocolate-5	0	8.9 ± 0.011	––
	10	18.8 ± 0.010	99.0 ± 1
	25	32.1 ± 0.012	92.8 ± 3

^a^Mean ± standard deviation

^b^*BDL* below detection limit

#### Comparison of the developed µ-SPE method with recent literature reports

The analytical performance of the developed CuMn₂O₄/Dy₂O₃-based µSPE method was benchmarked against previously reported solid-phase extraction strategies coupled with FAAS for Cd(II) determination. As summarized in Table 4, various magnetic and nanostructured adsorbents, such as Fe₃O₄@SiO₂-MIL-53(Fe), Co₃O₄@Fe₃O₄, Fe₃O₄@G2/Napht, Al–Fe₃O₄, and CaFe-LDH@g-C₃N₄, have been employed for trace cadmium extraction from diverse matrices, including water, food, and industrial samples. While the adsorbent dosages in these studies ranged from 5 to 200 mg, the proposed CuMn₂O₄/Dy₂O₃ nanocomposite required only 7.5 mg for efficient extraction. Moreover, the present method exhibited a superior limit of detection (LOD) of 5.2 µg·L⁻^1^, which is lower than or comparable to those reported in the literature. The developed procedure demonstrated excellent applicability to complex matrices, including municipal wastewater, juice, and commercial chocolate samples, highlighting its high sensitivity, low adsorbent consumption, and broad practical utility compared with previously reported methods. These comparisons confirm that the CuMn₂O₄/Dy₂O₃-based µSPE method provides a reliable, efficient, and environmentally favorable alternative for trace-level cadmium determination. However, the studies included in Table 4 were selected primarily from SPE/preconcentration methods coupled with FAAS in order to provide a comparison under relatively comparable operational conditions. Although ICP-MS- and GFAAS-based methods reported in the literature can achieve substantially lower detection limits, such techniques generally require more sophisticated instrumentation, higher operational costs, and greater maintenance demands compared with FAAS-based approaches.

**Table 4:** Comparison of the analytical performance of the developed CuMn₂O₄/Dy₂O₃-based micro-solid phase extraction method with previously reported adsorbents for Cd(II) determination

Adsorbent	Technique	Amount of adsorbent (mg)	LOD (µgL^−1^)	Real sample	Ref
Fe_3_O_4_@SiO_2_-MIL-53(Fe)	FAAS	20	13	Water, spice, chocolate, tea, and tobacco	(Soylak et al., 2023)
Co_3_O_4_@Fe_3_O_4_	FAAS	200	18	Seawater, tap water, mineral water, and bottled water	(Korkmaz et al., 2024)
Fe_3_O_4_@G2/Napht	FAAS	100	-	Tap water and River water	(Yetim et al., 2023)
Al-Fe_3_O_4_	FAAS	5	68	Green apple, red apple, Quince, Kiwi, Peanut, and Orange	(Ahmed et al., 2025)
CaFe-LDH@g-C₃N₄	FAAS	15	40	Spices, turmeric, Tap and Industrial wastewater	(Arain et al., 2025)
CuMn_2_O_4_/Dy_2_O_3_	FAAS	7.5	5.2	Wastewater, juice and commercial Chocolates	**Current Work**

## Conclusions

A novel CuMn₂O₄/Dy₂O₃ nanocomposite was successfully synthesized via a modified sol–gel method followed by thermal annealing and applied as an efficient adsorbent for micro solid-phase extraction (µSPE) of Cd(II) from complex matrices. Detailed physicochemical characterization confirmed its crystalline spinel structure, mesoporous morphology, high surface area, and thermal stability, all of which contributed to its superior adsorption performance. Optimization studies revealed that the extraction efficiency was highly dependent on pH, adsorbent dosage, contact time, eluent type, and sample volume. Under optimized conditions (pH 7.0, 7.5 mg of adsorbent, 30 s contact time, and 1.0 mL of 1 M HNO₃ as the eluent), the method achieved the maximum Cd(II) recovery. The analytical performance was outstanding, with an instrumental limit of detection of 5.2 µg·L⁻^1^, a linear dynamic range of 17.2–1900 µg·L⁻^1^, a preconcentration factor of 40, an enrichment factor of 39.4, and recovery rates above 98%, demonstrating high sensitivity, accuracy, and reproducibility. Application to certified reference materials and real samples, including municipal wastewater, juice, and commercially available chocolates, validated the method’s robustness and selectivity, while interference studies confirmed minimal matrix effects even in the presence of various coexisting ions. Compared with previously reported SPE-based Cd(II) extraction methods, the CuMn₂O₄/Dy₂O₃ adsorbent required less material and offered superior detection limits, highlighting both practical and analytical advantages. With a method LOD of 0.13 µg L⁻^1^ obtained by accounting for the preconcentration factor of 40, the procedure operates well below the WHO (3 µg L⁻^1^) and US EPA (5 µg L⁻^1^) drinking water guideline values for cadmium, making it suitable not only for food digests, wastewater, and juice samples but also for the routine monitoring of drinking and environmental waters. Overall, the developed µSPE–FAAS procedure provides a rapid, selective, and highly sensitive platform for the determination of trace amounts of cadmium in environmental and food matrices. Its excellent adsorption performance, coupled with minimal reagent consumption and low adsorbent mass requirement, underscores its suitability for routine analytical applications and its contribution to sustainable trace metal monitoring.

## Data Availability

All data analyzed during this study are included in this article. The raw data that support the findings of this study are available on request from the author.
